# Drug repurposing of natural depsipeptide from *Eleftheria terrae* isolated via iChip for anti-breast cancer therapy

**DOI:** 10.3389/fphar.2025.1694322

**Published:** 2025-10-27

**Authors:** Nuriyatul Fhatonah, Tedi Rustandi, Usama Ramadan Abdelmohsen, La Ode Akbar Rasydy, Ma’sum, Yomna Elghanam, Imran Pashar, Nordin, Ahmed Mahal, Akhmad Riski

**Affiliations:** ^1^ Department of Pharmacy, Universitas Muhammadiyah A.R. Fachruddin, Tangerang, Banten, Indonesia; ^2^ Department of Pharmacy, Sekolah Tinggi Ilmu Kesehatan ISFI Banjarmasin, Banjarmasin, Indonesia; ^3^ Deraya Center for Scientific Research, Deraya University, New Minia, Egypt; ^4^ Data Science, Evidence-Based and Clinical Research Laboratory, Department of Health, Social, and Clinical Pharmacy, College of Pharmacy, Chung-Ang University, Seoul, Republic of Korea; ^5^ Jurusan Keperawatan, Fakultas Kedokteran dan Ilmu Kesehatan, Universitas Lambung Mangkurat, Banjarmasin, Indonesia; ^6^ Guangzhou HC Pharmaceutical Co., Ltd, Guangzhou, China; ^7^ Master Pharmaceutical of Science, Faculty of Pharmacy, Universitas Gadjah Mada, Yogyakarta, Indonesia

**Keywords:** artificial intelligence, antibiotics, clovibactin, computational pharmacy, isolation chip, *in situ* culturing, microbial cultivation techniques, teixobactin

## Abstract

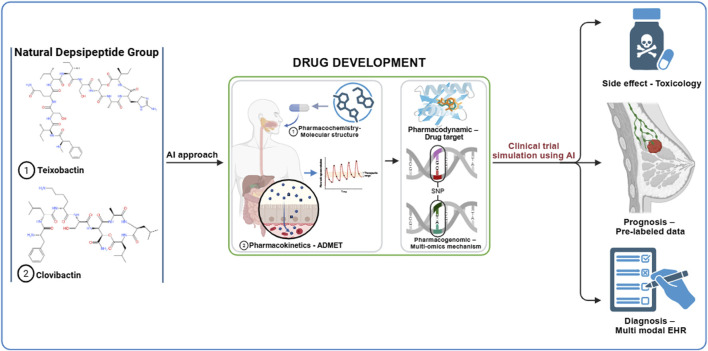

## 1 Introduction

In 2020, breast cancer was estimated to reach 2.3 million cases (new cases), with the impact of deaths occurring in the same year of 685,000 (Arnold et al., 2022). The total number of new cancer cases in women is approximately 9.2 million. Breast cancer accounts for approximately 2.3 million cases, representing approximately 24%–25% of all cancers in women. Breast cancer cases with high incidence rates are observed in Australia/New Zealand, Western Europe, North America, Central/South Asia, and parts of Africa. The highest breast cancer mortality rates are observed in Melanesia, Africa, and the Caribbean. New cases of breast cancer are expected to continue to increase, with a significant increase anticipated by 2050, resulting in a 38% increase (3.2 million new breast cancer cases) and causing 1.1 million breast cancer-related deaths annually (Kim et al., 2025).

Chemotherapy is the first-line treatment for breast cancer (Wang and Wu, 2023). Treatment regimens using this class of drugs have a better response profile and can prevent breast cancer (Gao et al., 2020). Treatment regimens using this class of drugs have a better response profile and can prevent breast cancer development (Davodabadi et al., 2023; Gu et al., 2025). A patient’s quality of life is affected by the side effects of chemotherapy, including neurotoxicity, gastrointestinal disturbances, cardiotoxicity, and immunosuppression (Brianna and Lee, 2023; Davodabadi et al., 2023; Kuderer et al., 2022; Was et al., 2022). However, the problem of chemotherapy drug toxicity remains a challenge, with suboptimal drug concentrations at the tumor site and significant systemic toxicity (Davodabadi et al., 2023; Raavé et al., 2018). Furthermore, the discovery and development of methods to reduce the limitations of breast cancer drugs require large investments and time (McIntosh et al., 2023; Michaeli et al., 2024). One solution to overcome this problem is to provide breast cancer drug repurposing that is effective, has minimal toxicity, and has low research costs.

A potential source of drugs that can be used as anti-breast cancer agents is bacterial secondary metabolites, such as *S. peucetius* var. Caesius, which produces doxorubicin (Bano et al., 2024; Rawat et al., 2021). Secondary metabolites derived from bacteria and their analogs have been repurposed as anti-breast cancer agents, including salinomycin, doxycycline, and tigecycline (Chen et al., 2022; Tang et al., 2017; Wang H. et al., 2021; Yu et al., 2024). Recent findings related to the therapeutic effects of antibiotics against breast cancer include those of benzimidazole derivatives and their derivatives (Lee et al., 2023). A benzimidazole derivative (BMPE) and prebiotic bacterial levan (LevAE) have been associated with triple-negative breast cancer (TNBC) in a 4T1-cell syngeneic mouse model (Shawky et al., 2025).

The high toxicity of antibiotic-derived breast cancer drugs should be addressed using drug repurposing approaches that explore other agents (Aggarwal et al., 2021; Kumbhar et al., 2022; Pfab et al., 2021). Currently, promising anticancer agents are those derived from natural depsipeptides isolated from *E. terrae* using an isolation chip (iChip). This technology produces new active metabolites with characteristics and activities that require further exploration (Chabib et al., 2025; Gauthier et al., 2025; Lodhi et al., 2018; Perrier et al., 2024). Antibiotics in this class include teixobactin and clovibactin, which are useful for treating infections caused by microbial resistance (Adeiza, 2024; Gunjal et al., 2020).

## 2 Natural depsipeptide characteristic

Natural depsipeptides, currently being developed as antibiotics, have the potential to be developed into anti-breast cancer agents. Natural depsipeptides, such as teixobactin and clovibactin, are a class of drugs that have peptide (amide) and ester bonds in their structure, particularly in their macrocyclic or linear structures. The structure of compounds in this class offers advantages, making them promising candidates for use as antibiotics against resistant bacteria owing to their ability to resist enzymatic degradation. Fragments that play a role in the structure of natural depsipeptides against enzymatic degradation are non-standard amino acids and structural motifs that have not been recognized or are not standard from existing structures. The stability of this drug against enzymatic degradation, as reported in previous studies, is strengthened by the presence of a bond between the d-amino acid and residues in the structure that possess macrocyclic properties (Chabib et al., 2025; Gunjal et al., 2020; Karas et al., 2020).

This class of drugs was first isolated from *E. terrae* bacteria, which were previously difficult to cultivate using traditional laboratory techniques. This class of drugs, including teixobactin and clovibactin, can only be cultured using methods known by several similar terms, including uncultured soil technology (UST), *in situ* incubation, and isolation chip (iChip) (Chabib et al., 2025). The presence of three d-amino acids and an l-allo-enduracididine residue distinguishes teixobactin from other natural peptides. The composition of teixobactin is believed to play a significant role in the action of this class of antibiotics against Gram-positive pathogens, such as methicillin-resistant *Staphylococcus aureus* (MRSA) and vancomycin-resistant *enterococci*. Its mechanism of action involves binding to lipids II and III, which affects bacterial cell wall synthesis. This mechanism causes significant bacterial cell death (Giltrap et al., 2016; Gunjal et al., 2020; Hussein et al., 2020; Karas et al., 2020).

A slightly different characteristic of teixobactin is its clovibactin compound, also known as Novo29. Clovibactin is the second compound after teixobactin that can be isolated from *E. terrae* using the iChip method. Clovibactin contains an eight-residue depsipeptide consisting of the rare amino acid hydroxy asparagine in a macrolactone ring. The structure of clovibactin, a macrolactone, enhances its antibacterial activity by binding to water and anions (Krumberger et al., 2023). The antibacterial activity of clovibactin against Gram-positive pathogens is similar to that of teixobactin, as it binds to the pyrophosphate groups of several essential peptidoglycan precursors (C55-PP, lipid II, and lipid III), thereby inhibiting bacterial cell wall synthesis (Adeiza, 2024; Shukla et al., 2023).

Modifications to natural depsipeptide compounds can enhance their antibacterial activities. Research has been conducted on teixobactin compounds, including modifications to the macrocyclic ring and N-terminal hydrophobic tail. This modification was performed by replacing l-allo-enduracidine with L-lysine. This modification can enhance the activity of these peptides against methicillin-resistant *Staphylococcus aureus* (MRSA) and vancomycin-resistant *enterococci* (VRE) (Morris et al., 2022; Parmar et al., 2018; Zong et al., 2019; 2018). Increased clovibactin activity was also observed in this study. Clovibactin modifications that can increase its antibacterial activity occur in the d-hydroxy asparagine fragment, followed by d-threonine, leucine, and cyclohexylalanine modifications (Adeiza, 2024; Bashir et al., 2024; Brunicardi et al., 2025; Shukla et al., 2022). Findings related to this modification can serve as the basis for modifying the structure of teixobactin or clovibactin into analogs that are effective as anti-breast cancer agents, with the aim of increasing efficacy and reducing toxicity.

## 3 Natural depsipeptide group as an anticancer agent

Teixobactin and clovibactin contain a cyclic tetra-depsipeptide bound to a depsipeptide, and four isoleucine residues are critical components of these compounds (Fiers et al., 2017; Matheson et al., 2019; Parmar et al., 2023). Compounds containing cyclic depsipeptides, such as Sansalvamide A exhibit anti-breast cancer activity *in vitro* in MDA-MB-231 cells (Trinidad-Calderón et al., 2023). These compounds inhibit histone deacetylase (HDACs) enzymes, including the Food and Drug Administration (FDA)-approved compounds romidepsin and vorinostat (El Omari et al., 2023; Lian et al., 2023; Parveen et al., 2023). Isoleucine in teixobactin is associated with anticancer activity by activating mTORC1. This activation slows cancer cell growth (Wang et al., 2023). However, these associations remain theoretical and should be approached with caution until validated by biochemical or cellular research. Further *in vitro* methodologies or simulations utilizing artificial intelligence may elucidate whether depsipeptides interact with HDAC or mTORC1 molecular targets.

The 13-membered macrolactone ring or cyclic tetra-depsipeptide contained in teixobactin and clovibactin has been previously studied and is closely related to their anticancer activity, including antimigratory, cytostatic, cytotoxic, and antiproliferative effects (Magpusao et al., 2010). Pharmacophores from this group can enhance antibacterial and anticancer activities by possessing amphipathic properties on hydrophilic and hydrophobic surfaces (Aghamiri et al., 2021; Gunjal et al., 2020; Shukla et al., 2022; Yang et al., 2020; 2017; 2016).

The potential of teixobactin as a breast cancer drug is supported by its good toxicity data. Teixobactin does not cause damage to human cells or eukaryotes. This mechanism is supported by data showing that teixobactin damages only lipid II-containing bacterial membranes (Matheson et al., 2019; Shukla et al., 2022). Clovibactin, which targets lipid II, has the same potential for low toxicity in both host and human cells (Sierra-Hernandez et al., 2025).

## 4 Future perspectives and research directions

While current research predominantly focuses on antibacterial activity, the distinctive capacity of these depsipeptides to target conserved molecular motifs and disrupt membrane-associated processes indicates their potential for anticancer applications. Numerous anticancer agents utilize analogous mechanisms, such as targeting the cell membrane integrity or biosynthetic pathways. Nevertheless, direct evidence or studies on teixobactin or clovibactin in cancer models have not been documented in the literature (Gunjal et al., 2020; Ling et al., 2015; Piddock et al., 2024; Shukla et al., 2023; 2020).

Structural modifications, including ongoing research on analogs and structure-activity relationships, may result in the development of derivatives with anticancer properties (Gunjal et al., 2020; Shukla et al., 2020). Mechanistic exploration necessitates Further studies are needed to determine whether the membrane-targeting actions of these depsipeptides can be effectively utilized against cancer cells. Drug discovery platforms, such as iChip technology and the investigation of uncultured bacteria, present new opportunities for identifying novel compounds with potential anticancer activity (Ling et al., 2015; Piddock et al., 2024).

## 5 AI-driven approaches

The drug repurposing of natural depsipeptide compounds, such as teixobactin and clovibactin, can be accelerated using an artificial intelligence (AI) approach at each stage of preclinical and clinical trials. The initial development of the AI approach involves creating large datasets that can be utilized for machine learning (ML) or deep learning (DL) analysis. The data required in the early stages include genomic, transcriptomic, and clinical databases. The expected results of the initial development are gene expression profiles, molecular pathways, and drug-target interactions (Bhinder et al., 2021; Issa et al., 2020; Tanoli et al., 2021; You et al., 2022; Zhou et al., 2023). Table 1 lists the AI tools relevant to the repurposing of anti-breast cancer drugs within the natural depsipeptide category. The graphical abstract outlines the AI-based drug repurposing process using natural depsipeptides as therapeutic agents for breast cancer. By combining rigorous hypothesis-driven research with advanced AI methodologies, future studies can more accurately determine whether depsipeptides, such as teixobactin and clovibactin, can evolve from antibacterial discoveries to validated anticancer applications.

**TABLE 1:** AI drug repurposing tools for natural depsipeptides as breast cancer agents. (1) Drug development and (2) Clinical trial simulation.

Stage	Tools	Function	References
(1)	a. SchNet4AIM b. Puchem c. ChemDB	Pharmacochemistry – Molecular structure	Gallegos et al. (2024), Rustandi et al. (2023), Wan et al. (2025)
(1)	a. TTD b. STITCH c. DrugBank d. AI-PBPK Modeling Platforms	Pharmacodynamic – Drug target	Rustandi et al. (2023), Vora et al. (2023), Wan et al. (2025), Wu et al. (2024)
(1)	a. DeepDRK b. Cmap c. KEGG d. TTD e. CCLE f. GDSC g. LINCS h. PathBank	Pharmacogenomic – Multi-omics mechanism	Wan et al. (2025), Wang Y. et al. (2021)
(1)	a. SwissADME b. AI-driven Drug Design (AIDD) Platform c. Neural Ordinary Differential Equations (Neural-ODE) d. Quantum Machine Learning (QML) e. ProTox 3.0	Pharmacokinetics - Absorption, Distribution, Metabolism, Excretion, and Toxicity (ADMET)	Bhatia et al. (2023), Chou and Lin (2023), Jones et al. (2024), Rustandi et al. (2025), Rustandi et al. (2023)
(2)	a. SIDER b. CTD c. Adverse Outcome Pathway (AOP) Modeling with AI	Side effect - Toxicology	Ciallella and Zhu (2019), Lin and Chou (2022), Rustandi et al. (2023), Wan et al. (2025)
(2)	a. Radiomics + AI b. OMIM c. CGI d. GtopDB e. AutoLDP (Automatic Labeling Tool)	Prognosis – Pre-labeled data	Bera et al. (2022), Sheth and Giger (2020), Wan et al. (2025), Zhao et al. (2025)
(2)	a. Multimodal fusion models (Early, Late, Hybrid) b. Generative adversarial networks (EHR-M-GAN)	Diagnosis – Multi-modal EHR	Li et al. (2023a), Li et al. (2023b), Liu et al. (2023), Mohsen et al. (2022), Tong et al. (2024)

## 6 Concluding remark

Breast cancer remains a global health challenge, with increasing incidence and mortality rates, necessitating the development of novel, effective, and low-toxicity therapeutics. The repurposing of natural depsipeptides, such as teixobactin and clovibactin, originally isolated from *E. terrae* using the iChip technique, offers a promising avenue for breast cancer therapy. These compounds exhibit unique structural properties, including macrocyclic depsipeptide motifs and non-standard amino acids, which confer resistance to enzymatic degradation and enhance their biological activity.

**FIGURE 1:**
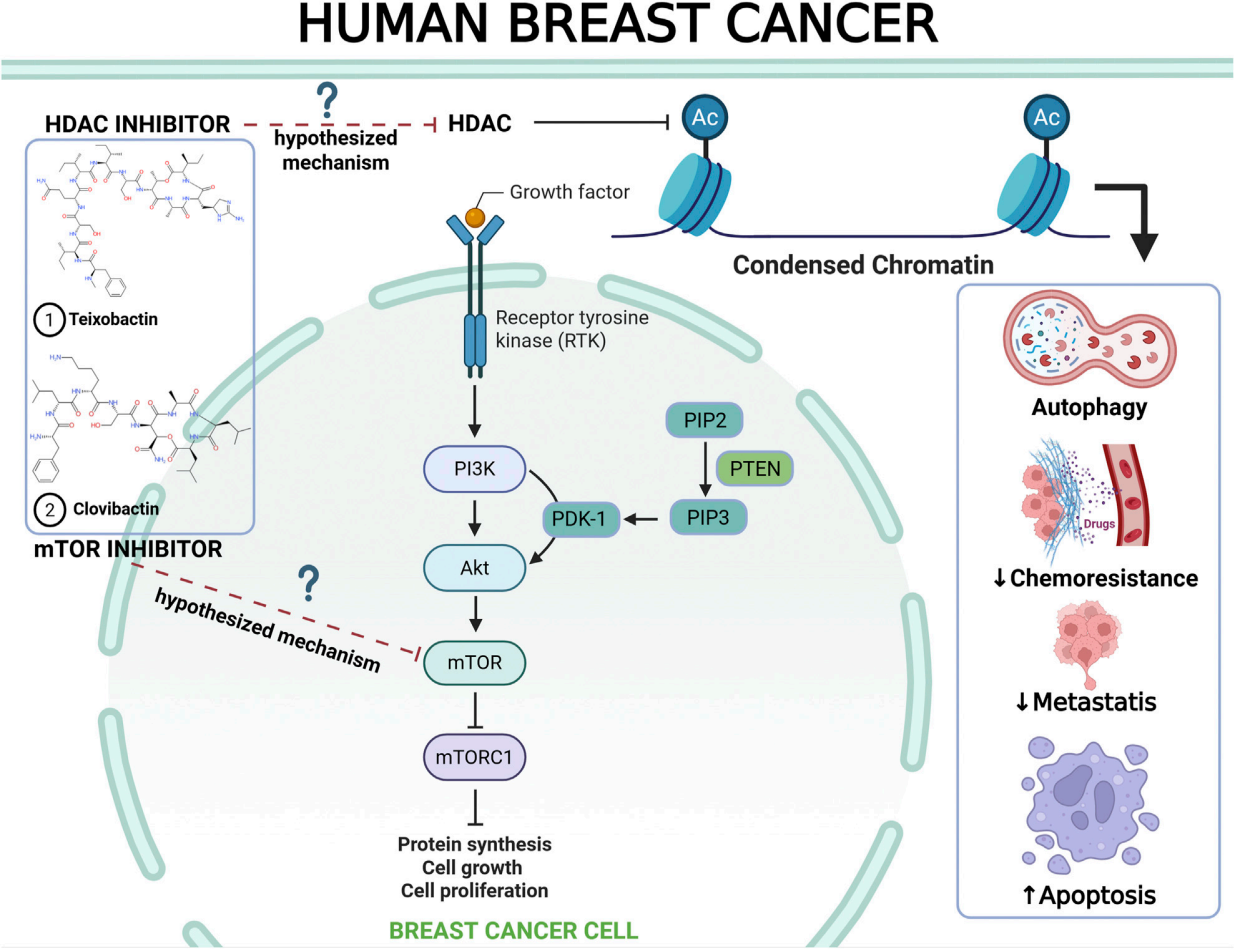
Proposed mechanistic hypothesis of natural depsipeptides (teixobactin and clovibactin) for anti-breast cancer therapy (Chilamakuri and Agarwal, 2022; El Omari et al., 2023; Khan et al., 2019; Parveen et al., 2023; Zucchetti et al., 2019).

Owing to their structural similarities, teixobactin and clovibactin are suggested to have anti-breast cancer effects, potentially through the inhibition of histone deacetylases (HDAC) and modulation of the mechanistic target of rapamycin complex 1 (mTORC1) pathway. Figure 1 depicts the proposed mechanism of action of the depsipeptide group, although these conceptual relationships require experimental validation. The integration of natural product discovery, AI-driven drug repurposing, and precision oncology has positioned natural depsipeptides as a groundbreaking therapeutic approach for breast cancer. Future research should focus on translational validation and combinatorial strategies to enhance clinical effectiveness. By harnessing advanced biotechnology and computational tools, this approach has the potential to revolutionize the standard of care for patients with breast cancer worldwide.

## 7 Limitations and hypothesis context

The proposed connection between teixobactin and clovibactin and their potential anticancer effects remain speculative and require further investigation. These ideas stem from the well-documented antibacterial properties of these substances and their structural resemblance to the known HDAC inhibitors. Therefore, this hypothesis should be approached with caution until supported by direct biochemical or cellular evidence. In conclusion, the proposed anticancer mechanisms for teixobactin and clovibactin should be regarded as theoretical and model-based extrapolations derived from their structural characteristics and antibacterial actions. These initial hypotheses offer a conceptual framework to guide future mechanistic research rather than providing definitive mechanistic assertions.
